# Polyuria in Sciatica

**Published:** 1910-07-09

**Authors:** 


### POLYURIA IN SCIATICA.

It is remarkable how many patients who suffer from sciatica also present polyuria, which begins with the pain of the sciatica, increases with it, and disappears as it subsides. If a second attack of sciatica should develop, polyuria is apt to occur again, though it may have entirely disappeared in the interval. The following are two cases illustrative of this point:—

The first was a vigorous man, fifty years of age, who had had an attack of sciatica three years previously, at which time the amount of urine he passed in twenty-four hours rose to between 100 oz. and 120 oz. A second attack of sciatica came on, and the polyuria, which had subsided in the interval, developed again; for a fortnight he passed daily 70 oz. to 132 oz. of urine, usually over 100 oz.

The second man was aged fifty-six years, who had had his first attack of sciatica, lasting three months, when he was fifty. Throughout that time he had been obliged to get up during the night to pass water two or three times, and this had not been the case either before or since until he developed a second attack of sciatica. He then passed 88 oz. in twenty-four hours, the composition of the urine being normal.

These observations might be multiplied, for although it can scarcely be said that every case of sciatica is associated with polyuria, it is true that a great many are so associated. The increase in the urine seems to be in some way reflex. It is noteworthy that whether the sciatica is simple, or due to interference with the sciatic nerve by a pelvic cancer, similar polyuria is apt to occur in either case.

